# A retrospective feasibility study of biweekly docetaxel in patients with high-risk metastatic castration-naïve prostate cancer

**DOI:** 10.1186/s12894-019-0463-7

**Published:** 2019-05-03

**Authors:** Sang Eun Yoon, Youjin Kim, Jangho Cho, Minyong Kang, Hyun Hwan Sung, Hwang Gyun Jeon, Byoung Chang Jeong, Seong Il Seo, Seong Soo Jeon, Hyun Moo Lee, Han Yong Choi, Su Jin Lee, Se Hoon Park

**Affiliations:** 1 Division of Hematology-Oncology, Department of Medicine, Samsung Medical Center, Sungkyunkwan University School of Medicine, 81 Irwon-ro, Gangnam-gu, Seoul, 06351 South Korea; 2 Department of Urology, Samsung Medical Center, Sungkyunkwan University School of Medicine, Seoul, South Korea; 3 Department of Urology, Kangbuk Samsung Hospital, Sungkyunkwan University School of Medicine, Seoul, South Korea

**Keywords:** Castration-naïve prostate cancer, Docetaxel, Biweekly

## Abstract

**Background:**

Results from randomized phase III trials have shown that thrice-weekly docetaxel added to androgen-deprivation therapy (ADT) has a significant impact on the survival of patients with metastatic castration-naïve prostate cancer (mCNPC) and established early chemotherapy as part of the standard of care for high-risk disease. Controversy remains, however, because some patients experience critical toxicities related to docetaxel. The purpose of the current study was to evaluate the feasibility and adverse events of biweekly-administered docetaxel in patients with previously-untreated, high-risk mCNPC.

**Methods:**

The study included 35 consecutive patients with high-risk mCNPC who received ADT plus docetaxel 40 mg/m^2^. Oral prednisone 5 mg twice daily was also given. Treatment was repeated every two weeks for up to 12 cycles or until disease progression or unacceptable toxicity occurred. High-risk was defined as bone metastases beyond axial skeleton and/or visceral disease.

**Results:**

The included patients’ median age was 68 years (range: 31–86 years) and 17 (49%) had visceral metastases. Biweekly docetaxel was generally well-tolerated; the most commonly observed adverse events, considering those of all grades, included alopecia (74%), nail changes (42%), and constipation (31%). Hematologic adverse events were infrequent, and no patient received hematopoietic growth factors. One patient died after the fourth cycle due to respiratory failure, which occurred as a complication of pneumonia. Among the 35 patients, 28 completed the planned 12 cycles of biweekly docetaxel. Prostate-specific antigen response (> 50% decrease from baseline) was recorded in 33 patients (94%), and the radiologic response rate was 49%. Median progression-free survival was 13.6 months (95% confidence interval: 6.7–20.4).

**Conclusion:**

ADT plus biweekly-administered docetaxel appeared to be tolerated and effective in patients with high-risk mCNPC.

## Background

Prostate cancer is one of the greatest growing cancers in Korea, with an estimated 9785 new cases and 1667 deaths occurring in Korea in 2014 [1]. For patients with metastatic or advanced disease, androgen-deprivation therapy (ADT), which includes bilateral orchiectomy or medical castration with gonadotropin-releasing hormone (GNRH) agonists, can provide palliation of symptoms and prolong survival [2]. If a patient develops castration-resistant prostate cancer (CRPC), docetaxel is regarded as the standard first-line chemotherapy regimen from the results of two randomized clinical trials [3, 4].

Docetaxel is commonly administered at a dose of 75 mg/m^2^ every 3 weeks established by the results from the TAX-327 study [5, 6], in which once every 3 weeks docetaxel (median: 19.2 months, 95% CI, 17.5 to 21.3 months) conferred a definite survival benefit over weekly docetaxel for 3 weeks (median: 17.8 months, 95% CI, 16.2 to 19.2 months).

In Asian countries, there was better survival benefit in every 3 weeks administration(12.5 months) than every week administration (8.0 months). No grade 3 or 4 neutropenia was showed in once weekly for 3 weeks, but Gr 3/4 neutropenia was observed more frequently about 75% of patients with CRPC who were treated with once every 3 weeks docetaxel [7]. Based on previous studies, a pharmacokinetics study conducted in Japan [8] and consideration of efficacy and toxicities in the management of solid tumors in a palliative setting [9], docetaxel is most commonly administered in Korea and Japan at a lower dose (i.e., 60 mg/m^2^ every 3 weeks).

An additional option to prevent docetaxel-related hematologic toxicity is to use other administration schedules, such as weekly or biweekly regimens. On the side of this approach, biweekly administration of docetaxel 50 mg/m^2^ showed a better time-to-failure (TTF; median: 5.6 months vs. 4.9 months; *P* = 0.014) than thrice-weekly docetaxel [10]. As expected, the biweekly regimen was better tolerated than the thrice-weekly docetaxel regimen, and importantly, efficacy was not compromised. In our own retrospective study [11], we showed that a docetaxel 40 mg/m^2^ biweekly regimen was appropriate and well-tolerated in Korean patients with CRPC.

Although surgical or medical castration is considered standard treatment in castrate-naïve prostate cancer (CNPC), some patients with extensive metastatic (i.e., high-risk) disease at diagnosis, including visceral or bone involvement beyond the axial skeleton, have shorter survival times [12]. It is not enough to start standard hormone castration treatment alone to high tumor volume at CNPC diagnosis. The previous randomized phase III trials were conducted using either six or nine cycles of docetaxel 75 mg/m^2^ every 3 weeks in the CNPC setting [13–15]. Their results led to early change to ADT in high-risk CNPC in most guidelines published by the oncology societies [16, 17]. Supposedly, the new standard therapy should only be applied in patients fit enough to receive docetaxel and tolerate the associated side effects. Based on these considerations, from October 2013, we adopted a biweekly low-dose docetaxel regimen (40 mg/m^2^ every 2 weeks) as an institutional standard chemotherapy regimen for patients with chemotherapy-naïve CNPC. The present study was conducted to evaluate the tolerability of the regimen and to obtain feasibility data in anticipation of performing a prospective, formal phase II study.

## Methods

We retrospectively collected and reviewed the medical records of 35 men with metastatic CNPC who were consecutively treated with biweekly docetaxel and ADT as first-line therapy between October 2015 and December 2016. In the present study, we included the patients with histologically-confirmed adenocarcinoma of the prostate and naïve to both ADT and chemotherapy. The other eligibility criteria consisted of an Eastern Cooperative Oncology Group (ECOG) performance status of 0 or 1, radiologic and clinical evidence of high-risk metastatic disease for which there was no curative treatment available, and acceptable major organ functioning to receive chemotherapy. High-risk was defined as bone metastases beyond the axial skeleton and/or visceral disease, regardless of prostate-specific antigen (PSA) level.

The choice of early docetaxel was determined by a multidisciplinary urologic oncology team composed of urologists, radiologists, pathologists, radiation oncologists, and medical oncologists. The study protocol was approved by the Samsung Medical Center (Seoul, Korea) Institutional Review Board, and all patients provided written informed consent prior to undergoing docetaxel chemotherapy.

### Treatment

Our medical center standard chemotherapy for CRPC has been a biweekly docetaxel 40 mg/m^2^ regimen [11], corresponding to a dose intensity of 20 mg/m^2^/week (equivalent to 60 mg/m^2^ every 3 weeks). Docetaxel was administered intravenously over 1 hour on day 1 with dexamethasone and antiemetics. Oral prednisolone 5 mg was administered twice daily from day 1 and continued throughout treatment. Supportive care, including the administration of blood products, bisphosphonates, and the use of analgesics, was given if judged appropriate by the treating physicians. Prior to initiation of the first dose of docetaxel, a complete history was taken from each patient. In addition, complete blood counts, serum chemistry analyses, chest X-rays, bone scans, and computed tomography (CT) scans of all involved sites were performed. Patients were seen every 2 weeks; during these visits, a brief history was taken; a physical examination was performed; and adverse events, blood counts, and PSA level were assessed. Treatment was repeated on an outpatient basis and continued until objective disease progression, unacceptable toxicity, patient refusal, or up to 12 cycles. Throughout the treatment period, ADT with GNRH agonists was given to all participants, except those who underwent surgical castration. Radiologic responses were evaluated every 8 weeks by bone scan, chest and abdominopelvic CT, or the same tests that were used for initial tumor staging. Adverse events were collected and graded according to the National Cancer Institute’s Common Terminology Criteria for Adverse Events version 4.

### Statistical analysis

The evaluation of toxicity was the primary endpoint for the study, and myelosuppression, specifically grade 3 or 4 neutropenia and febrile neutropenia, was considered to be the dose-limiting toxicity. Other endpoints included a PSA response, defined as a ≥ 50% decline in PSA level from baseline with no clinical or radiologic evidence of disease progression, overall survival (OS), and progression-free survival (PFS). PFS was defined as the time from the first administration of docetaxel to the date of disease progression (PSA or radiologic progression) or death. PSA progression was defined as an increase ≥25% and ≥ 2 ng/mL after 12 weeks according to the Prostate Cancer Working Group 2 criteria [18]. PFS and OS were calculated using the Kaplan–Meier method. All analyses were performed using R for Windows, version 2.11.1 (http://www.r-project.org; R Core Team, Vienna, Austria).

## Results

The baseline patient characteristics of 35 patients who were registered between October 2015 and December 2016 are listed in Table 1. Their median age was 68 years (range: 31–86 years), and 71% had symptomatic (i.e., ECOG performance status of 1) disease. The most commonly involved metastatic sites were bone (97%) and lymph nodes (91%), and 17 participants (49%) had visceral metastases. ADT was initiated within 1 month of starting docetaxel in all patients in combination with leuprolide (57%), goserelin (40%), or degarelix (3%).

**Table 1:** Patient characteristics

	No.	%
Age, years		
Median (range)	68 (31–86)	
Disease status		
Metastatic at diagnosis	31	89
Recurrent	4	11
Gleason score		
6	1	3
7	5	14
8	13	37
9	12	34
10	4	11
Initial PSA, ng/mL		
Median (range)	330.08 (4–50,000)	
Type of metastases		
Bone and/or lymph nodes only	18	51
Visceral	17	49
Metastatic site		
Lymph node	32	91
Bone	34	97
Lung	16	46
Liver	4	11
ECOG performance status		
0	10	29
1	25	71

*PSA* prostate-specific antigen, *ECOG* Eastern Cooperative Oncology Group

Because the patients could have metastases at multiple sites, the total number of metastases cases is greater than the number of patients

The included patients received a total of 1483 cycles of biweekly docetaxel (median: 12, range: 1–12). Three patients discontinued treatment after the first cycle, two due to toxicity or withdrawal and one due to disease progression. Docetaxel was delayed in 17 cycles (1%), and dose reduction was required in five patients (14%). As a dose intensity of docetaxel 20 mg/m^2^/week was planned, the relative dose intensity was 98% [95% confidence interval (CI): 86–104]. Biweekly docetaxel showed manageable toxicities including all grades (Table 2). The most ordinary adverse events were alopecia (74%), nail changes (42%), and constipation (31%). Hematologic adverse events presented rarely. There were five patients required blood transfusion due to anemia and/or thrombocytopenia, but they did not receive hematopoietic growth factors. One patient died of respiratory failure induced pneumonia complication after completing the fourth cycle chemotherapy.

**Table 2:** Maximum grade of toxicities per patient

	Grades 1/2 N (%)	Grades 3/4 N (%)
Anemia	8 (22.9)	4 (11.4)
Neutropenia	6 (17.1)	0
Thrombocytopenia	4 (11.4)	1 (2.9)
Alopecia	26 (74.3)	0
Fatigue	6 (17.1)	0
Nausea	4 (11.4)	0
Anorexia	5 (14.3)	0
Peripheral neuropathy	8 (22.9)	0
Stomatitis	7 (20)	0
Diarrhea	3 (8.6)	1 (2.9)
Constipation	11 (31.4)	0
Skin	9 (25.7)	0
Nail changes	15 (42.9)	0

There was no complete clinical response in the current study. 31 patients evaluated for radiologic responses and 17 patients (55, 95% CI: 46–64) presented partial responses. and 10 patients achieved stable disease. Finally, 87% of the overall patients obtained adequate disease control.

**Fig. 1:**
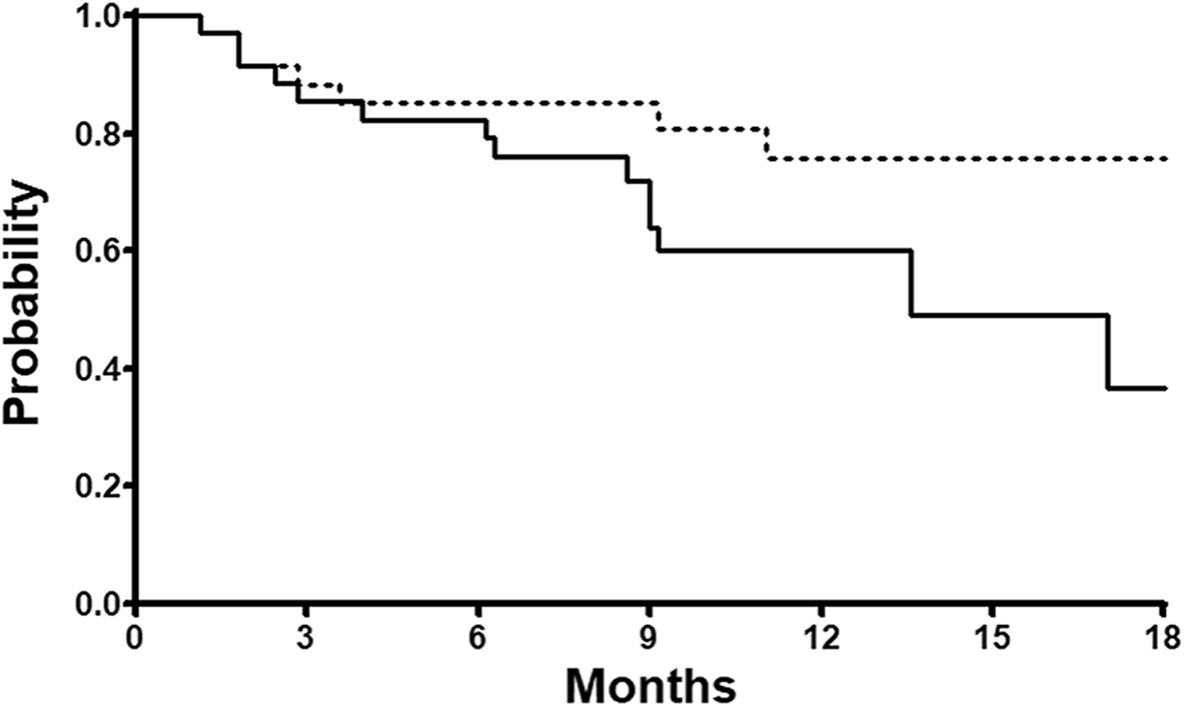
Kaplan–Meier curve for PFS (solid line) and OS (dotted line)

Thirty-three patients (94%) demonstrated PSA response (> 50% decrease from baseline), and 49% patients appeared radiologic response. Median PFS was 13.6 months (95% CI: 6.7–20.4). Median OS was not reached, but the OS rate at 1 year was identified as 75% (Fig. 1).

## Discussion

There are newly developed numerous treatment options for advanced prostate cancer, but it is not yet possible to replace all of the docetaxel [17]. The standard docetaxel regimen (75 mg/m^2^ every 3 weeks plus prednisone) should significantly improve overall survival for long time in metastatic CRPC, but more often grade 3–4 adverse events. In the current study, we reported that docetaxel 40 mg/m^2^ every 2 weeks plus prednisolone was similar in efficacy and manageable toxicities compared to previous studies.

Docetaxel administrated 75 mg/m2 every 3 weeks is mostly used at the standard of chemotherapy of mCRPC. However, therapy-related adverse effects and treatment related mortality were prevalent in elderly and fragile patients to receive standard regimen. Although the response of docetaxel 75 mg/m2 every 3 weeks schedule was favorable, it did not show the good overall survival for these patients. [19].

In a retrospective study, we evaluated the feasibility of biweekly docetaxel 40 mg/m2 and compared with 75 mg/m2 every 3 weeks for mCRPC. Their median age was 68 years (range, 52–84). The patients received biweekly docetaxel 40 mg/m2 presented similar TTP (5.0 months vs 4.2 months, *p*-value = 0.530) and minor incidence of Gr 3/4 adverse events. This result exhibited one of the treatment schedule options for mCRPC [11].

There were no comparable studies of biweekly docetaxel 40 mg/m2 and 75 mg/m2 every 3 weeks for mCNPC. In the CHAARTED study, the researchers compared the efficacy and toxicity of docetaxel (at a dose of 75 mg/m^2^ every 3 weeks for 6 cycles) plus ADT and ADT alone of 790 mCNPC patients. The docetaxel plus ADT regimen demonstrated better outcomes than ADT alone (57.6 months vs. 44.0 months; *P* < 0.001) in patient with mCNPC. The current outcome was not equally comparable with the CHAARTED due to the follow-up period was shorter, as so we need a long-term follow-up period to present the outcome including OS. However, adequate response rate reached 87% even in the short observation period, we considered one of the treatment options.

We already know that the high tumor burden is a poor prognostic factor. Each clinical study had also dealt with the correlation survival and high tumor burden. In the subgroup analysis from the CHAARTED study, a high volume of metastases was defined by the presence of visceral metastases or four or more bone lesions with at least one beyond the vertebral bodies and pelvis. There were 57 (14.4%) patients with visceral metastasis who had poor median OS (49.2 months) than in all CNPC patients (57.6 months) [13, 14]. In the GETUG-AFU15 study, the median OS was approximately 62.1 months in the mCNPC patients, and less than 36 months in the mCNPC patient with high-volume, high-risk disease [14].

In current study, we included that all patients had high-volume CNPC such as visceral metastasis (49%) and bone metastasis (51%). The result (median PFS: 13.6 months, 95% CI: 6.7–20.4) was not as bad as expected from high risk. However, it is limited in interpretation of the PFS because of small sample size and short follow up period. Based on recent randomized trials [13–15] and our data, the addition of docetaxel to ADT is recommended for patients with high-risk, metastatic CNPC who are medically-fit enough to tolerate docetaxel.

In the CHAARTED, the total dosage of docetaxel 75 mg/m^2^ every 3 weeks for 6 cycles was 450 mg, and the period of administration was 18 weeks. Similarly, the total dosage of the 40 mg/m^2^ every two-week regimen was 480 mg, and the period of administration was 24 weeks. There was a little difference in docetaxel dosage or administration period between the two regimens. Nevertheless, it showed a different pattern of drug toxicities between the two studies. Approximately 12.1% of patients in the CHAARTED had grade 3 or 4 neutropenia and 6.1% had grade 3 or 4 febrile neutropenia. [13, 14]. In present study, the most common hematologic toxicity of any grade was anemia (*n* = 12), and grade 3 or 4 hematological adverse events occurred infrequently. There was no big distinctness in the dose and the toxicity was lower in 40 mg/m^2^ every two-week regimen. So, we could suggest A as other administration schedules on these results.

The major limitations of our retrospective study include small sample size and lack of a control arm to better define differences between biweekly dosing and standard dosing in this population. Further accumulation of cases with longer follow-up periods is necessary. Within the limitations of our study, we demonstrated that early chemotherapy with biweekly docetaxel regimen resulted in tolerability and activity in patients with high-risk CNPC.

ADT plus biweekly-administered docetaxel appeared to be tolerated and effective in patients with high-risk mCNPC comparable with CRPC. Our results suggest that a biweekly docetaxel chemotherapy regimen is considered to have manageable toxicities and to yield acceptable results compared to a thrice-weekly docetaxel regimen.

## Conclusion

In conclusion, ADT plus biweekly-administered docetaxel appeared tolerable toxicities and similar efficacy compared with standard thrice-weekly docetaxel. From this result, we could suggest that docetaxel 40 mg/m2 biweekly regimen is an additional treatment option for mCNPC.

## Abbreviations

ADT: Androgen-deprivation therapy
CNPC: Castration-naïve prostate cancer
CRPC: Castration-resistant prostate cancer
GNRH: Gonadotropin-releasing hormone
